# Fasting and surgery timing (FaST) audit

**DOI:** 10.1016/j.clnu.2020.08.033

**Published:** 2021-03

**Authors:** Ahmed M. El-Sharkawy, Prita Daliya, Christopher Lewis-Lloyd, Alfred Adiamah, Francesca L. Malcolm, Hannah Boyd-Carson, Daniel Couch, Philip J.J. Herrod, Tanvir Hossain, Jennifer Couch, Panchali B. Sarmah, Tanvir S. Sian, Dileep N. Lobo, FaST Audit Group, Shahira Anjum, Opusdei Aghanenu, Sarah Barlow, Wosu Chukwuemeka, Jennifer Couch, Prita Daliya, Hamid Daud, Rebecca Green, Tanvir Hossain, Michael King, Anisa Kushairi, Dileep N. Lobo, Thomas Moreno-Stokoe, Ashrafun Nessa, Olamide Oyende, Adil Rashid, Jack Starkie, Alfred Adiamah, David R. Andrew, Amanda Koh, Christopher Lewis-Lloyd, Farah Roslan, Sona Singh, Amari Thompson, Chris Busby, Ahmed M. El-Sharkawy, Sita Kotecha, Javed Latif, Kevin Sargen, Hannah Boyd-Carson, Daniel Couch, Phillip J.J. Herrod, Jonathan N. Lund, Francesca L. Malcolm, Jonathan M. Pourrie, Siddhee Pradhan, Nanin Rai, Tanvir S. Sian, Muhammed J. Al-Ausi, Andrew Fitzsimonds, Ashwini Ghorpade, Ashish Kelkar, Panchali B. Sarmah, James Wolff, the East Midlands Surgical Academic Network (EMSAN)

**Affiliations:** f Nottingham University Hospitals NHS Trust, UK; g United Lincolnshire Hospitals NHS Trust, UK; h Chesterfield Royal Hospitals NHS Foundation Trust, UK; i University Hospitals of Derby and Burton NHS Foundation Trust, UK; j Kettering General Hospital NHS Foundation Trust, UK; a East Midlands Surgical Academic Network, Queen's Medical Centre, Nottingham, NG7 2UH, UK; b Gastrointestinal Surgery, Nottingham Digestive Diseases Centre and National Institute for Health Research (NIHR) Nottingham Biomedical Research Centre, Nottingham University Hospitals and University of Nottingham, Queen's Medical Centre, Nottingham, NG7 2UH, UK; c Department of Surgery, University Hospitals of Derby and Burton NHS Foundation Trust, Uttoxeter Road, Derby, DE22 2QG, UK; d Leicester Cancer Research Centre, University of Leicester, Leicester, LE2 7LX, UK; e MRC Versus Arthritis Centre for Musculoskeletal Ageing Research, School of Life Sciences, University of Nottingham, Queen's Medical Centre, Nottingham, NG7 2UH, UK

**Keywords:** Preoperative fasting, Elective surgery, Emergency surgery, Adherence to guidelines, Food, Clear liquids

## Abstract

**Background & aims:**

International guidance advocates the avoidance of prolonged preoperative fasting due to its negative impact on perioperative hydration. This study aimed to assess the adherence to these guidelines for fasting in patients undergoing elective and emergency surgery in the East Midlands region of the UK.

**Methods:**

This prospective audit was performed over a two-month period at five National Health Service (NHS) Trusts across the East Midlands region of the UK. Demographic data, admission and operative details, and length of preoperative fasting were collected on adult patients listed for emergency and elective surgery.

**Results:**

Of the 343 surgical patients included within the study, 50% (n = 172) were male, 78% (n = 266) had elective surgery and 22% (n = 77) underwent emergency surgery. Overall median fasting times (Q1, Q3) were 16.1 (13.0, 19.4) hours for food and 5.8 (3.5, 10.7) hours for clear fluids. Prolonged fasting >12 h was documented in 73% (n = 250) for food, and 21% (n = 71) for clear fluids. Median fasting times from clear fluids and food were longer in the those undergoing emergency surgery when compared with those undergoing elective surgery: 13.0 (6.4, 22.6) *vs*. 4.9 (3.3, 7.8) hours, and 22.0 (14.0, 37.4) *vs*. 15.6 (12.9, 17.8) hours respectively, p < 0.0001.

**Conclusions:**

Despite international consensus on the duration of preoperative fasting, patients continue to fast from clear fluids and food for prolonged lengths of time. Patients admitted for emergency surgery were more likely to fast for longer than those having elective surgery.

## 1 Introduction

Traditionally, patients have been kept ‘nil-by-mouth’ for 12–24 h prior to elective surgical procedures in order to mitigate the risk of vomiting and aspiration at the time of induction of anaesthesia [1]. Although this practice lacked a supporting evidence base, it was often recommended as it was easy for hospital staff and patients to follow and understand. However, there is now evidence supporting the benefits of shorter preoperative fasting times whilst maintaining a low risk of aspiration during induction of anaesthesia [[2], [3], [4]]. This is supported by human physiology studies demonstrating that clear liquids are emptied from the stomach within 2 h after ingestion with an approximate gastric emptying time of 4 h for food, depending on the type and quantity ingested [[5], [6], [7]].

In view of the evidence, modern national and international guidelines currently recommend that all patients undergoing non-emergency surgery requiring a general anaesthetic fast for 2 h from clear fluids and 6 h from food [[8], [9], [10], [11]]. However, anecdotal and published evidence suggests that there are widespread variations in the duration for which patients are fasted preoperatively, with many patients subjected to much longer periods of preoperative fasting [[12], [13], [14], [15]]. In 2011 Falconer et al. [12] audited elective preoperative fasting times in a large UK centre and reported that patients were fasted for a median (Q1, Q3) of 9.4 (5.4, 12.8) and 13.5 (11.5, 16.0) hours from fluids and food respectively. Prolonged fasting times of 13.0 (8.5, 16.2) and 17.4 (13.7, 28.5) hours from fluids and food respectively in patients undergoing emergency surgery [13]. Similar prolonged preoperative fasting times have also been reported from other centres [14,15].

Prolonged fasting is not only difficult for patients to tolerate; it is also associated with dehydration [16,17]. Dehydration, particularly in older adults has been linked with acute kidney injury (AKI) which is associated with poor outcomes [18,19]. Preoperative fasting also induces metabolic stress leading to the depletion of glycogen stores, the utilisation of muscle protein for gluconeogenesis and the development of postoperative insulin resistance [20]. Insulin resistance and consequent hyperglycaemia may be of clinical significance due to its association with increased infective complications, morbidity, length of hospital stay and mortality [[21], [22], [23], [24]]. However, the evidence for reduction in postoperative complications by providing preoperative carbohydrate drinks is debatable [25,26]. Prolonged fasting from clear fluids has also been shown to complicate the induction of anaesthesia, with patients fasted for prolonged periods having a significantly lower blood pressure during induction within a paediatric population [4].

This multicentre study aimed to audit preoperative fasting times prospectively in patients undergoing planned (elective) and emergency general surgical operations with a primary objective of assessing adherence to current guidelines on preoperative fasting.

## 2 Methods

The audit was intended as a snapshot of practice and was conducted between 1st March 2018 and 30th April 2018. Patients listed for surgical intervention were triaged by the FaST Study Group daily at five National Health Service (NHS) Trusts across the East Midlands region of the United Kingdom; including two of the UK's largest teaching hospitals (Table 1).

**Table 1:** Description of National Health Service (NHS) Trusts where the data were collected.

NHS Trust	Day only bed numbers	Overnight bed numbers
Nottingham University Hospitals NHS Trust	187	1647
University Hospitals of Derby and Burton NHS Foundation Trust	106	1465
United Lincolnshire Hospitals NHS Trust	215	1199
Kettering General Hospital NHS Foundation Trust	38	672
Chesterfield Royal Hospital NHS Foundation Trust	–	536

Bed availability and occupancy data – Total number of available day only and overnight beds between January and March 2018 as listed on NHS England website: https://www.england.nhs.uk/statistics/statistical-work-areas/bed-availability-and-occupancy/bed-data-day-only/.

Inclusion criteria consisted of adult patients ≥16 years of age admitted to surgical departments for emergency or elective operations, including patients planned for overnight admission and same day discharge after surgery. Patients admitted to theatre directly from an acute care environment, National Confidential Enquiry into Patient Death and Outcome (NCEPOD) “immediate” classification, and those undergoing reoperations during the same admission were excluded [27]. Patients were recruited preoperatively on the day of surgery and patient reported data were collected in the form of a questionnaire. A variety of operations from a number of surgical subspecialties were included under the remit of general surgery. These included the surgical divisions of upper gastrointestinal (oesophagogastric and hepatopancreaticobiliary), colorectal, endocrine, breast, general and emergency general, and vascular surgery. Non-general surgical specialties contributing data included urology, plastic surgery, oral and maxillo-facial, and ear, nose and throat surgery. Prospective data collected included patient demographics, admission and operative details, and information on the patients’ last preoperative meal and drink.

Operations were defined using the NCEPOD classification of surgical intervention [27]. Elective operations were defined as those planned prior to patient admission. Emergency operations, in accordance with the UK National Emergency Laparotomy Audit (NELA), were defined as non-elective procedures for conditions that posed a potential threat to life that were classed as either urgent or expedited requiring surgery within hours to days of the decision to operate [28]. This definition is in congruence with previous large epidemiological studies defining emergency surgery as non-elective surgery within 48 h of admission [29]. The type of anaesthesia was categorised into general, regional, or local. Surgery type was divided into the general surgical subspecialties: *‘upper gastrointestinal’*, *‘colorectal’* and *‘general’* surgery with all other surgical procures classed as *‘other’*. Preoperative fasting times were measured in hours from last drink or meal to the induction of anaesthesia. The surgical approach was defined as either laparoscopic, majority of the operation was completed using a minimally invasive technique, or open, the operation was performed using an open or converted from a laparoscopic approach. Only one NHS Trust had a pre-existing system in place to improve preoperative hydration (the “Think Drink” campaign, a nationally recognised scheme [30,31]) in elective surgical patients prior to and during the conduct of this study.

### 2.1 Ethics and permissions

As this was a clinical audit reviewing existing service provision, formal ethical approval was not required. The study was registered with the local audit office at each participating site, and audit and information governance standards were adhered to. Although written consent was not obtained, patients were informed of the audit and were actively involved to obtain information on fasting details.

### 2.2 Data analysis

Anonymised data from each centre were collated with all data management and analyses performed using Stata® version 16.0 (StataCorp LLC, College Station, Texas, USA). Data were assessed for normality visually by viewing distribution plots. Normally distributed data were presented as means and standard deviations (SD). Non-parametric data were presented as medians and interquartile ranges (Q1, Q3) with the Mann Whitney U test used to assess statistical significance. Chi squared analysis was used to assess statistical significance between categorical variables. P values < 0.05 were considered statistically significant, with confidence intervals calculated at 95%. Where possible missing data were fitted as a separate category or declared in all analyses. Multivariable linear regression was used to analyse the effect of independent variables on preoperative fasting times from clear fluids and food. Normalisation of data was achieved by taking the natural logarithm (ln(x)) of the non-parametric dependant variables. Measures of effect were obtained by calculating the natural exponential (e^x^) of the regression coefficient (β) to formulate a ratio effect measure (e^β^). P values presented in the multivariable analysis are derived from the likelihood ratio test. Within subgroup analysis elective morning operating lists were scheduled between 08:00 and 13:00, afternoon lists were scheduled from 13:00 until 17:00 with elective operations categorised either as day case or requiring an overnight admission.

## 3 Results

**Fig. 1:**
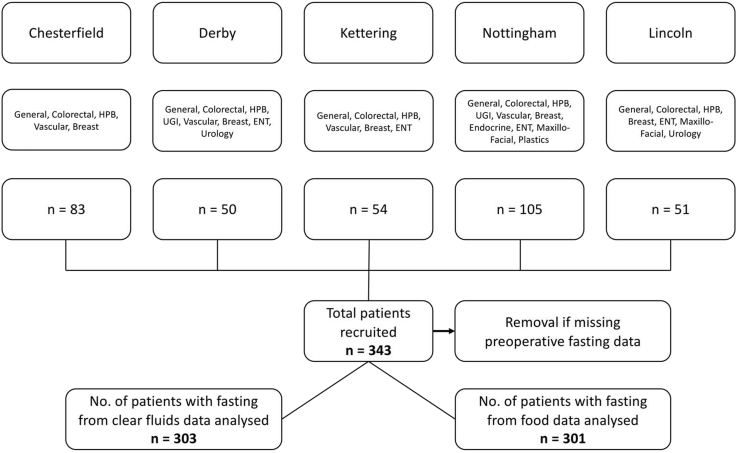
Study flow diagram.

A total of 343 patients (Fig. 1) were included in the study, of whom 77 (22%) underwent emergency and 266 (78%) elective operations. Of the elective patients 80 (30%) underwent day case operations where patients were discharged on the day of surgery. Table 2 lists patient characteristics. The overall median (Q1, Q3) fasting times for food and clear fluids were 16.1 (13.0, 19.4) and 5.8 (3.5, 10.7) hours, respectively. All patients undergoing elective surgical procedures were sent written information outlining recommended preoperative starvation times, of these, 190 (71%) and 191 (72%) reported the recommended fasting times accurately from clear fluids and food respectively. The median (Q1, Q3) perceived allowed fasting times reported by the patients were 11.2 (8.0, 18.8) hours for food and 5.7 (3.2, 9.0) hours for clear fluids.

**Table 2:** Patient demographics.

		All Patients (n = 343)	Emergency Patients (n = 77)	Elective Patients (n = 266)
Age (years) - Mean (SD)		55.6 (18.0)	48.0 (19.7)	57.8 (16.9)
Gender - n (%)	Male	172 (50%)	38 (49%)	134 (50%)
	Female	171 (50%)	39 (51%)	132 (50%)
Body mass index kg/m^2^- Median (Q1, Q3)^a^		27.6 (24.0, 31.8)	28.8 (22.9, 35.7)	27.4 (24.0, 31.4)
Anaesthetic type - n (%)	General	314 (92%)	75 (97%)	239 (90%)
	Local/Regional	17 (5%)	1 (1%)	16 (6%)
	Missing	12 (4%)	1 (1%)	11 (4%)
Type of surgery - n (%)	Upper GI	54 (16%)	3 (4%)	51 (19%)
	Colorectal	77 (22%)	18 (23%)	59 (22%)
	General	117 (34%)	49 (64%)	68 (26%)
	Other	80 (23%)	4 (5%)	76 (29%)
	Missing	15 (4%)	3 (4%)	12 (5%)
Surgical approach - n (%)	Laparoscopic	123 (36%)	32 (42%)	91 (34%)
	Open	204 (59%)	41 (53%)	163 (61%)
	Missing	16 (5%)	4 (5%)	12 (5%)
Preoperative fasting time (hours) - Median (Q1, Q3)	Clear fluids^b^	5.8 (3.5, 10.7)	13.0 (6.4, 22.6)	4.9 (3.3, 7.8)
	Food^c^	16.1 (13.0, 19.4)	22.0 (14.0, 37.4)	15.6 (12.9 17.8)
Received intravenous fluids pre-operatively	Yes	80 (23%)	48 (62%)	32 (12%)
	No	263 (77%)	29 (38%)	234 (88%)
Patient perceived allowed preoperative fasting time (hours) - Median (Q1, Q3)	Clear fluids^d^	5.7 (3.2, 9.0)	10.6 (6.5, 21.9)	4.7 (3.0, 7.3)
	Food^e^	11.2 (8.0, 18.8)	15.0 (10.2, 37.4)	10.2 (7.9, 14.3)

a Number of patients in analysis: All (303), Emergency (56), Elective (247).

b Number of patients in analysis: All (303), Emergency (69), Elective (234).

c Number of patients in analysis: All (301), Emergency (67), Elective (234).

d Number of patients in analysis: All (253), Emergency (56), Elective (197).

e Number of patients in analysis: All (257), Emergency (58), Elective (199).

**Fig. 2:**
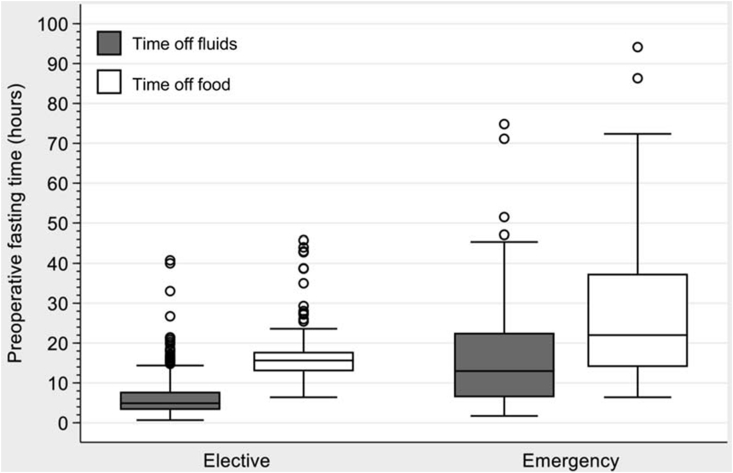
Box plots showing preoperative fasting times for fluids and food. p < 0.0001 for fluids *vs*. food and for elective *vs*. emergency surgery.

**Fig. 3:**
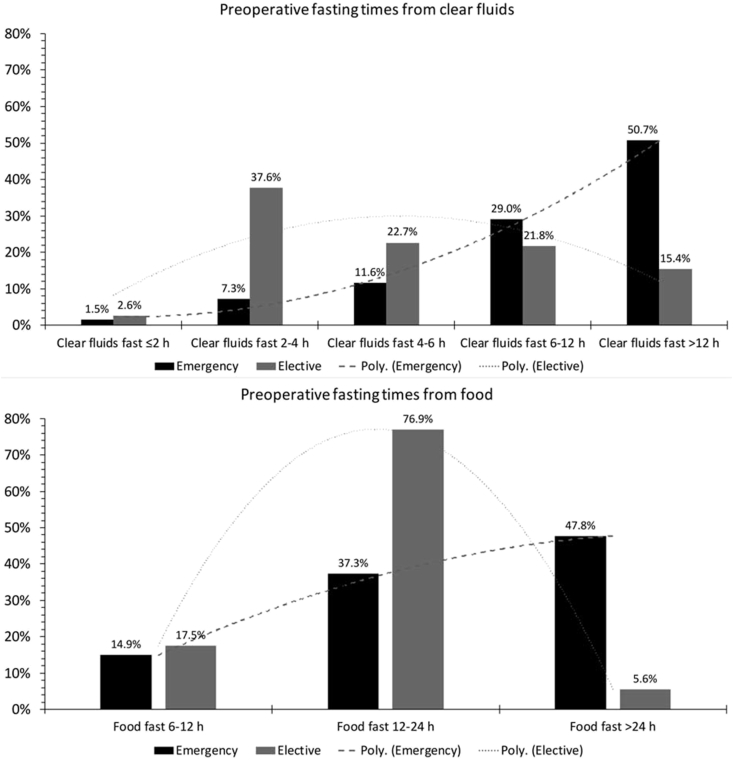
Variation in proportion of elective and emergency patients according to duration of fasting from clear fluids and food. (Poly. = polynomial trend line).

Substantially prolonged fasting of >12 h from food and clear fluids was noted in 73% and 21% of all cases respectively with fasting for >24 h from food noted in 13%. Prolonged starvation was much more frequent in patients undergoing emergency surgery when compared with those having elective surgery. The distribution of preoperative fasting times from clear fluids and food for patients undergoing elective and emergency surgery are described in Fig. 2. The median (Q1, Q3) fasting times from food were higher in the emergency than elective group: 22.0 (14.0, 37.4) hours *vs*. 15.6 (12.9, 17.8) hours respectively, p < 0.0001. Fasting times from clear fluids was also significantly longer in the emergency compared with the elective group: 13.0 (6.4, 22.6) *vs*. 4.9 (3.3, 7.8) hours respectively, p < 0.0001. Figure 3 demonstrates the variation in proportion of elective and emergency patients according to duration of fasting from clear fluids and food respectively. The majority of the elective patients (38%) fasted from clear fluids for between 2 and 4 h. Whereas the minority of patients undergoing emergency surgery (7%) fasted from clear fluids for 2–4 h, the majority (51%) fasted for >12 h, p < 0.0001 trend. The majority of elective patients (77%) fasted from food for between 12 and 24 h, with a minority (6%) fasting for >24 h. However, the majority of emergency patients (48%) fasted from food for >24 h with a minority (15%) fasting for between 6 and 12 h, p < 0.0001 trend.

On multivariable analysis of preoperative fasting times there were significant differences between elective and emergency patients fasting from both clear fluids and food. Emergency patients were found to fast from clear fluids more than twice as long as elective patients [adjusted e^β^ 2.15 (1.67, 2.77), p < 0.0001] (Table 3) and fast from food 36% longer than elective patients [adjusted e^β^ 1.36 (1.18, 1.56), p < 0.0001] (Table 4). Age, sex, BMI and administration of intravenous fluid were not found to significantly affect preoperative fasting times.

**Table 3:** Unadjusted and adjusted ratios of the natural logarithm of preoperative fasting time from clear fluids.

	Unadjusted exponential of regression coefficient (*e*^β^) (n = 303)	95% Confidence intervals		p value	Adjusted exponential of regression coefficient (*e*^β^) (n = 272)^a^	95% Confidence intervals		p value
Admission type (emergency)	2.28	1.88	2.76	<0.0001	2.15	1.67	2.77	<0.0001
Age (years)	0.99	0.99	1.00	0.01	1.00	0.99	1.00	0.40
Sex (male)	1.02	0.85	1.22	0.83	1.09	0.92	1.30	0.31
Body mass index (kg/m^2^)	1.01^b^	0.99	1.02	0.21	1.00	0.99	1.02	0.52
Intravenous fluids preopertaively (yes)	1.51	1.23	1.85	0.0001	1.02	0.81	1.28	0.87

All p values from likelihood ratio test.

a Adjusted for all other variables in table.

b 272 patients in analysis.

**Table 4:** Unadjusted and adjusted ratios of the natural logarithm of preoperative fasting time from food.

	Unadjusted natural logarithm of regression coefficient (*e*^β^) (n = 301)	95% Confidence intervals		p value	Adjusted natural logarithm of regression coefficient (*e*^β^) (n = 269)^a^	95% Confidence intervals		p value
Admission type (emergency)	1.47	1.32	1.65	<0.0001	1.36	1.18	1.56	<0.0001
Age (years)	1.00	1.00	1.00	0.60	1.00	1.00	1.00	0.21
Sex (male)	1.02	0.92	1.12	0.74	0.99	0.90	1.09	0.90
Body mass index (kg/m^2^)	1.00^b^	0.99	1.01	0.73	1.00	0.99	1.00	0.35
Intravenous fluids preoperatively (yes)	1.35	1.21	1.51	<0.0001	1.07	0.94	1.22	0.30

All p values from likelihood ratio test.

a Adjusted for all other variables in table.

b 272 patients in analysis.

On subgroup analysis of elective patients, the median (Q1, Q3) fasting time from food for when scheduled for the morning operating list was 15.3 (12.7, 17.3) compared with 16.6 (15.0, 18.7) hours for the afternoon session of the operating list, p = 0.07. The median (Q1, Q3) fasting time from clear fluids for elective cases when scheduled for the morning operating list was 4.5 (3.2, 8.0) compared with 5.5 (3.5, 7.6) hours for the afternoon session of the operating list, p = 0.3. No statistical differences were observed between the fasting times from clear fluids between patients undergoing elective surgery where the “Think Drink” initiative was established or not, median 4.4 (3.1, 7.3) and 5.2 (3.3, 8.0) hours respectively, p = 0.12. This was also observed when fasting from food, median 15.6 (13.1, 17.7) *vs*. 15.8 (12.6, 18.0) hours respectively, p = 0.82. However, patients who underwent elective surgery in the morning where “Think Drink” was established fasted from clear fluids for significantly shorter durations than those where the initiative was not present, median 4 (3, 5.8) *vs*. 5 (3.3, 8.8) hours respectively, p = 0.02. This was not observed in afternoon elective lists. Within the emergency patient subgroup 38% did not receive intravenous fluids, with 37% of patients fasting from clear fluids for >12 h failing to receive preoperative intravenous fluids.

## 4 Discussion

This multicentre prospective audit from the East Midlands region of the UK demonstrates poor compliance with national and international preoperative fasting guidelines. Patients were fasting from clear fluids and food much longer than is recommended, with the majority of patients undergoing elective surgery fasting >4 h from clear fluids and >12 h from food. Patients undergoing emergency surgery fasted for even more prolonged periods with the majority fasting >12 h from clear fluids and >24 h from food.

This audit has several strengths. First, the study took place in multiple centres, in both tertiary and district general hospitals, with a variety of surgical subspecialties contributing data. This variety reduces the selection bias attributed to single centre studies and reduces the effect of intra-hospital guidelines on outcome data and so aids the generalisability of the results to the wider surgical patient population. Secondly, the inclusion of patients undergoing elective and emergency surgery allows for the comparison of different preoperative surgical pathways and their effect on patient optimisation before surgery. Thirdly, the exclusion of emergency patients admitted directly to theatre from an acute clinical area, where fasting rules do not necessarily apply, ensured the accuracy and validity of the data presented was not overly skewed by this cohort of patients. However, the significant proportion of missing data within this study may affect the results and by reducing the sample size for analysis increasing both selection bias and the possibility of type II errors. Although data were collected prospectively, there was reliance on patient participation and patient recall, with additional potential problems with bias. This study was unable to address whether patients who fasted for prolonged time periods had significantly different postoperative outcomes when compared with patients fasted in accordance with current guidance. Further research specifically looking at postoperative outcomes and length of preoperative fasting is required to address this.

These findings are similar to previously published works from single centres with smaller sample sizes [[12], [13], [14], [15]] and go against the principles of modern perioperative guidelines [[8], [9], [10], [11]], including the enhanced recovery after surgery (ERAS) protocols [32]. The present audit also demonstrates that a significant proportion of patients are fasting for over 12 h before surgery. This practice is likely to be associated with significant patient discomfort as well as thirst and hunger. Moreover, prolonged fasting may lead to dehydration and insulin resistance, resulting in poor clinical outcome. A meta-analysis investigated the association between preoperative fasting and complications in patients undergoing laparoscopic cholecystectomy [33] with the authors reporting increased postoperative comfort, improved insulin resistance and a reduced stress response associated with shortened preoperative fasting times. This was also associated with a reduction in postoperative nausea and vomiting (lg(odds ratio [OR]), −0.24; 95% CI, −0.48 to 0.00; P = 0.046). The authors also reported that prolonged preoperative fasting times were associated with high rates of nausea and vomiting in the first 24 h after surgery that affected the patients' postoperative recovery and resulted in longer hospital stay [33].

The reasons for prolonged preoperative fasting times are likely to be multifactorial. These range from poor staff understanding to challenges with efficiency and scheduling, which therefore impair compliance with guidelines [34]. Patient education, understanding and engagement may also play a key role as demonstrated from the findings of this audit where a significant proportion of patients reported prolonged preoperative fasting times despite receiving written information detailing this. Similar findings have been reported in previous studies which demonstrated that when patients were allowed unlimited clear fluids until 3 h before the operation, only 40% consumed fluids after midnight [34]. These findings may be, in part, due to some patients not reading or understanding the information, but may also be a manifestation of stress and anxiety that can reduce the ability of patients to comply [35]. Another important consideration is that whilst the information advises patients on suggested preoperative fasting guidelines, wording can often be ambiguous and difficult to interpret. For example, previous versions of preoperative fasting guidelines stated patients should fast for “at least” 2 h (instead of “no more than”) which can be misinterpreted by patients to suggest longer fasting times may be preferable [36]. Many guidelines have been altered to eliminate such ambiguity including the European guidelines on preoperative fasting that state adults should be encouraged to drink clear fluids up to 2 h before elective surgery [8,36]. In addition, the use of both verbal and written instructions in the elective setting is likely to result in better compliance rather than written communication alone [37]. It also important to note whilst most patients scheduled for elective operations will receive written information, they are rarely offered an explanation justifying preoperative fasting. This may also contribute to poor compliance as poor patient understanding has been demonstrated to result in a five-fold decrease in compliance [38] and is supported by the social learning theory that states “patients are more likely to follow physicians’ instructions if they believe that: (a) they are susceptible; (b) noncompliance could lead to a problem with serious effects; (c) they have the knowledge that enables them to avoid the problem; and (d) compliance to the instructions will reduce the risk of complications” [39,40]. Poor patient information and education may, therefore, result in noncompliance. Many of these factors may be addressed using pre-existing infrastructure, facilitating cost effective ways for improvement. Schiff et al. [41] reported that patients who were seen at an anaesthesia preoperative clinic demonstrated better knowledge of why they needed to fast preoperatively than those who were seen on the ward (84.0% *vs*. 59.0%; p < 0.001). This may translate to better compliance.

Another factor that may have contributed to resistance to the uptake of the more liberal guidelines on preoperative fasting has been concerns about the complexity of the advice which may result in misunderstanding and cancelled operations. However, an investigation on the effect of the ‘new’ nil by mouth policy that allowed unrestricted intake of clear fluids up to 3 h preoperatively found no change in cancellations of operations, theatre utilisation or efficiency [34].

The present audit also demonstrated that patients generally fasted for much longer from food than clear fluids. This is in part a reflection of the guidelines that suggest longer fasting time from food than clear fluids but this may also be influenced by the reluctance of patients to wake early to eat particularly when scheduled for a morning operation and reduced appetite associated with stress and anxiety. Interestingly, however, we did not find any significant differences in fasting times between patients scheduled for elective morning and those scheduled for afternoon lists. Preoperative carbohydrate loading, where carbohydrate drinks are administered the evening before and 2 h before surgery, have been suggested as an effective way to mitigate this and attenuate the development of insulin resistance [20,42]. However, the clinical importance of these carbohydrate drinks on postoperative morbidity is debatable. A recent randomised controlled trial showed there was no difference in postoperative infective complications between patients given preoperative carbohydrate treatment and controls, although those who received carbohydrate drinks required less insulin to control postoperative blood glucose concentrations [26]. A systematic review and meta-analysis also found that carbohydrate drinks did not reduce postoperative complications [25]. Despite this uncertainty, carbohydrate loading regimens are now incorporated into ERAS programs which aim to mitigate the catabolic state associated with surgery and to aid enhanced recovery [32].

Fear of aspiration is also important to consider as some anaesthetists still advise prolonged preoperative starvation in order to mitigate aspiration at induction of anaesthetic. Murphy et al. investigated this and reported no episodes of aspiration as well as fewer rapid-sequence induction/intubations or awake intubations performed after introduction of the ‘new guidelines’ [34].

Given the persistent poor compliance with preoperative fasting guidelines demonstrated in this study and results published by others from the UK, Europe and across the world, a renewed multidisciplinary approach is required with patients at its heart. Patients and all members of the multidisciplinary team need to be aware of the aims and goals of preoperative fasting and participate in clear and effective communication and feedback. Newton et al. [43] demonstrated significant improvement in compliance with preoperative fasting guidelines after using systematic quality improvement methodology to assess and implement meaningful change. They developed many interventions with an opt out scheme where the surgeon and anaesthetists opt patients out where prolonged preoperative fasting is required. Some of the key concepts introduced included allowing the nursing team to give patients a drink on arrival to the hospital given that most patients did not arrive in theatre within an hour presenting to hospital for elective operations, this intervention resulted the most significant improvement. Other centres have introduced successful campaigns such as ‘Think Drink’ which have introduced algorithms for staff to improve communication and cohesion between theatres, wards and admission areas that have shown improvement in compliance with guidelines [30,31]. These studies all aimed to address the major themes identified in the EXPERIENCE Study [44] that investigated the barriers to implementing current best clinical practice in perioperative nutrition. The study found “complexity of the context”, e.g. required flexibility and unpredictable theatre times, hierarchical decision making with lack of confidence in junior staff to implement change and poor teamwork and communication as the main reasons for defaulting to habitual behaviour. However, as demonstrated by another study [45], the notion that implementing a new evidence-based protocol is sufficient to drive change is inadequate. Experience, that comes with time, and improved organisation of care on a multidisciplinary level are required in order for this to occur [45].

Better utilisation of existing data on operative times, theatre efficiency and organisation may help improve the scheduling and planning of preoperative fasting in both emergency and elective settings. Putting the patients at the heart of any changes and providing better information as well as empowering them by explaining the reasoning for the guidelines will likely result in better understanding, increased compliance and improved clinical outcomes.

## Funding

This work was supported by the 10.13039/501100000265Medical Research Council [grant number MR/K00414X/1]; and 10.13039/501100000341Arthritis Research UK [grant number 19891]. AA was funded by a 10.13039/501100000272National Institute for Health Research Academic Clinical Fellowship.

The funders had no role in the design or conduct of the work, or in the decision to publish. This paper presents independent research funded by the 10.13039/501100000265MRC, 10.13039/501100000341ARUK and the 10.13039/501100000272NIHR. The views expressed are those of the authors and not necessarily those of the MRC, ARUK, NHS, the NIHR or the Department of Health.

## Author contributions

Study design: AME-S, PD, AA, HBC, DC, PJJH, TH, JC, PBS, TSS, DNL.

Development of questionnaire: AME-S, PD, AA, HBC, DC, PJJH, TH, JC, PBS, TSS, DNL.

Data collection: FaST Audit Group.

Data-analysis: CL-L, AA, AME-S.

Data-interpretation: AME-S, PD, CL-L, AA, DNL.

Writing of Manuscript: AME-S, PD, CL-L, AA, FLM, PJJH, DNL.

Creation of figures: CL-L, AA, DNL.

Critical review of Manuscript: AME-S, PD, CL-L, AA, PJJH, DNL.

Final approval: AME-S, PD, CL-L, AA, FLM, HBC, DC, PJJH, TH, JC, PBS, TSS, DNL.

## Conflicts of interest

None of the authors has a direct conflict of interest to declare. DNL has received an unrestricted research grants for unrelated work from B. Braun in the last 3 years. He has also received speakers’ honoraria from B. Braun, Fresenius Kabi, Shire and Baxter Healthcare for unrelated work in the last 3 years.

## Appendix

Shahira Anjum, Opusdei Aghanenu, Sarah Barlow, Wosu Chukwuemeka, Jennifer Couch, Prita Daliya, Hamid Daud, Rebecca Green, Tanvir Hossain, Michael King, Anisa Kushairi, Dileep N Lobo, Thomas Moreno-Stokoe, Ashrafun Nessa, Olamide Oyende, Adil Rashid, Jack Starkie (Nottingham University Hospitals NHS Trust); Alfred Adiamah, David R Andrew, Amanda Koh, Christopher Lewis-Lloyd, Farah Roslan, Sona Singh, Amari Thompson, (United Lincolnshire Hospitals NHS Trust); Chris Busby, Ahmed M El-Sharkawy, Sita Kotecha, Javed Latif, Kevin Sargen (Chesterfield Royal Hospitals NHS Foundation Trust); Hannah Boyd-Carson, Daniel Couch, Phillip J J Herrod, Jonathan N Lund, Francesca L Malcolm, Jonathan M Pourrie, Siddhee Pradhan, Nanin Rai, Tanvir S Sian (University Hospitals of Derby and Burton NHS Foundation Trust); Muhammed J Al-Ausi, Andrew Fitzsimonds, Ashwini Ghorpade, Ashish Kelkar, Panchali B Sarmah, James Wolff (Kettering General Hospital NHS Foundation Trust).
